# The Effect of Using Micro-Clustered Water as a Polymer Medium

**DOI:** 10.3390/ijms22094730

**Published:** 2021-04-29

**Authors:** Żaneta Król-Kilińska, Dominika Kulig, Ihar Yelkin, Anna Zimoch-Korzycka, Łukasz Bobak, Andrzej Jarmoluk

**Affiliations:** 1 Department of Functional Food Products Development, The Faculty of Biotechnology and Food Science, Wroclaw University of Environmental and Life Sciences, Chelmonskiego 37/41, 51-630 Wroclaw, Poland; dominika.kulig@upwr.edu.pl (D.K.); anna.zimoch-korzycka@upwr.edu.pl (A.Z.-K.); lukasz.bobak@upwr.edu.pl (Ł.B.); andrzej.jarmoluk@upwr.edu.pl (A.J.); 2 Plasma Investment Ltd., Research and Development Department, Dunska 13, Wroclaw Technological Park, 54-427 Wroclaw, Poland; i.yelkin@plasmainvestment.com

**Keywords:** water, micro-clustered, sodium alginate, carrageenan, gelatin, rheology, hydrosol, plasma

## Abstract

The aim of the study was to investigate the changes within the physicochemical properties of gelatin, carrageenan, and sodium alginate hydrosols prepared on the basis of micro-clustered (MC) water. The rheological parameters, contact angle and antioxidant activity of hydrosols were investigated. Moreover, the pH, oxidation–reduction potential (ORP) and electrical conductivity (EC) were measured. The hydrosols with MC water were characterized by a lower pH, decreased viscosity, a lower contact angle, and only slightly lower antioxidant activity than control samples. The results showed that hydrosol’s properties are significantly changed by MC water, which can lead to enhancement of its applicability but requires further investigation.

## 1. Introduction

Plasma is considered the fourth state of matter, consisting of electrons, negative and positive ions, atoms, free radicals, molecules in both the ground and excited state and UV photons [1]. Two types of plasma can be distinguished: thermal (hot) and non-thermal (cold) plasma, depending on the average energies of the plasma particles and their relevant degrees of freedom. In recent years, non-thermal plasma has been of interest to many scientists. The temperature of the cold plasma is less than 60 °C, making it suitable for many applications. Plasma generates ions, ozone (O_3_), UV photons, and various reactive oxygen and nitrogen species (RONS), which makes it a potential tool for food decontamination, water purification, and the sterilization of medical tools and packaging materials [2]. Moreover, plasma has shown a promising effect in plant growth promotion, pest control, toxin removal, agrochemical dissipation, cancer therapy, tissue regeneration, etc. [3]. However, a few researchers revealed that applying the gas plasma directly over the food surface can cause loss of color and degradation of bioactive compounds [4]. One of the ways to avoid deterioration in quality is to use micro-clustered water (MC), also known as plasma-treated water, plasma acid, or plasma activated liquids [1]. Water molecules form a hydrogen-bonded network consisting of clusters. Water clusters have been proposed as an explanation of the unusual physical characteristic of water, such as its high boiling/freezing point, the abnormal temperature dependency of its density, and ice nucleation for freezing [5]. The structure of water changes significantly if ions, neutral molecules or gas are introduced to it. According to Yelkin et al. [6], plasma treatment causes disintegration of larger water agglomerates into smaller ones, affecting its properties. MC contains significant amounts of chemically active species produced in plasma and at the plasma–liquid interface, which leads to its acidification. The pH and RONS concentration of MC depends on the type of used electrodes, plasma source and power, formed plasma volume, time of plasma generation, initial water chemistry and water volume [7]. The active ingredients and lower pH of MC make it a versatile medium for biomedical and agricultural applications [8]. The antimicrobial activity of MC results from the presence of RONS, which causes damage to the cell membrane, cell wall and DNA, cell shrinkage and cytoplasmic leakage. Some of the studies have focused on the use of MC as a plant disinfectant [3], food disinfectant [9,10,11], and inducer for plant growth [12].

Plasma reactors can be classified into three groups based on the following: direct discharges in liquids; discharges in the gas phase over a liquid; and discharges in bubbles inside liquids, contacting liquid spray mist or foams. The principle of operation of these reactors is based on the transfer of ions, electrons and radicals to the surface of the treated material. However, plasma treatment of liquids can involve contact or non-contact interactions. The first may produce free radicals produced by immediate interactions of the liquid molecules with electrons and ions in plasma while the non-contact are mostly based upon coupling of the liquids with the plasma generated spectrum, which is in reach of both infrared and ultraviolet spectral lines, which excite the species within a liquid. A novel plasma treatment that was recently proposed [6] was used in this study in which the plasma–liquid interaction is based on wideband noise generated in plasma discharge, which, luckily, plays the role of an efficient noise-transmitting antenna. In the latter case, which we have adopted, the liquid is contained in dielectric vessels, which are non-transparent to visual and ultraviolet radiation. This is because the noise generated in plasma lies in the range of acoustic frequencies up to tens of MHz. An increase in the strength of vibrations in water clusters may cause breaking of the hydrogen bonds connecting ions into clusters and, therefore, lead to changes in the structure of the water.

Although there is much literature on the use of MC, there are no studies on its use as a solvent for polymers. Polymers are widely used in a variety of industrial sectors due to their biodegradability, non-toxicity, biocompatibility and bioactivity. They have several functions, such as thickening and gelling, stabilizing foams, inhibiting crystal formation, etc. [13]. They are used as edible coatings or films to provide control of moisture loss, microbiological stability, the controlled release of flavors to the product, etc. [14]. For the purpose of this study, there were three different polymers chosen with the ability to form gels: sodium alginate, gelatin and carrageenan. Sodium alginate is a natural anionic polysaccharide composed of linear chains of 1–4 linked α-l-guluronic (G) and β-d-mannuronic (M) acid residues with free hydroxyl (OH^−^) and carboxylate (-COO^−^) groups distributed along the backbone. It is extracted from various species of brown algae [15]. Sodium alginate has been used successfully in the food, pharmaceutical, and cosmetic industries as packaging material, dispersion and emulsion stabilizer, wound dressing material, matrix for capsules and microcapsules, etc. [16,17,18]. Carrageenan is a marine-derived polysaccharide composed of β-d-galactose and α-d-galactose linked through β-(1,4) and α-(1,3) links. Due to its superior gelling and high viscosity properties, it is commonly used in the food industry as a thickening, stabilizing, and suspending agent [19]. Gelatin is obtained from the irreversible thermal degradation of collagen. It shows excellent gelling, swelling and surface properties, essential for films, hydrogels and emulsions preparation. Gelatin is widely used for active packaging, microencapsulation and the production of medicines [20].

The aim of the study was to evaluate the possibility of using micro-clustered water for hydrosol preparation. We hypothesize that the use of MC results in hydrosols’ physiochemical and rheological changes, leading to enhancement of its applicability.

## 2. Results and Discussion

### 2.1. Physiochemical Properties of Hydrosols

**Figure 1:**
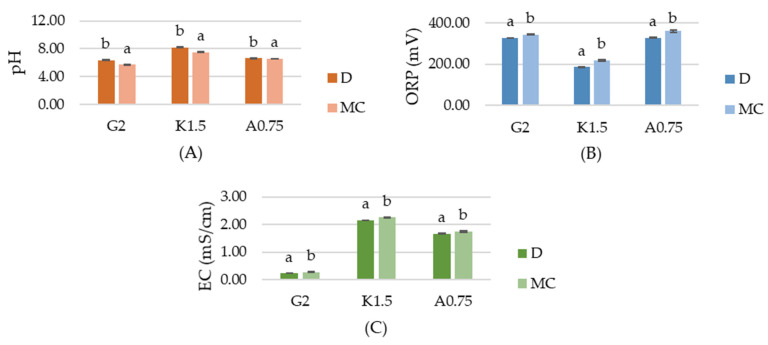
The effects of using plasma activated water on the (**A**) pH, (**B**) oxidation–reduction potential (ORP), (**C**) conductivity (EC) of gelatin (G2), carrageenan (K1.5), sodium alginate (A0.75) hydrosols. ^a,b^ Different letters indicate significantly different groups determined by Duncan’s test (*p* < 0.05).

The physiochemical properties of the tested hydrosols, including pH, oxidation–reduction potential (ORP) and conductivity (EC), are shown in Figure 1. The pH of gelatin, carrageenan and sodium alginate hydrosols with micro-clustered water (MC) was significantly lower than with distilled water (D) and was equal to 5.71, 7.51, 6.54, respectively. According to Shaw et al. [2], non-thermal plasma (NTP) generates ions, ozone (O_3_), UV photons and reactive oxygen and nitrogen species (RONS), such us O (atomic oxygen), OH (hydroxyl radical), O_3_ (ozone), H_2_O_2_ (hydrogen peroxide), NO (butric oxide), NO^2−^ (nitrites), NO^3−^ (nitrates) and ONOO^−^ (peroxynitrates). A decrease in pH of hydrosols with MC can be caused by the nitric and nitrous acid formed from the reactive species generated during plasma discharge. The increased EC of the MC hydrosols confirms active ions accumulation. Compared to the D hydrosols, the EC of gelatin, carrageenan and sodium alginate MC hydrosols was higher by 15%, 5.1% and 4.1%, respectively. ORP (oxidation–reduction potential) is a measurement of electron activity that indicates the oxidizing or reducing potential of a medium. The lowest ORP was measured for G2D, K1.5D and A0.75D hydrosols, while the ORP values of MC hydrosols were significantly higher and equal to 343 mV, 218 mV, 360 mV, respectively, which suggests the generation of reactive chemical species in MC hydrosols.

### 2.2. Rheological Measurements

#### 2.2.1. Flow Property

Results of the shear flow properties of gelatin, carrageenan and sodium alginate hydrosols are shown in Table 1. The Ostwald de Waele model and the Hershchel–Bukley model fitted the experimental data well. The results show that the flow properties of the tested materials are dependent on the polymer concentration and the type of water (D or MC). A significant increase (*p* < 0.05) in the viscosity values at a 5 s^−1^ shear rate ($\dot{\gamma })$ with the increase of polymer concentration was noticed. The apparent viscosity (**η**) of polymer solutions appears as a continuous function of their molecular weight [21]. According to this statement, the increased polymer concentration related to a number of polymer molecules per unit volume is a leading cause of viscosity incrementation. The use of MC water for carrageenan and sodium alginate hydrosols preparation caused a significant decrease in apparent viscosity at a constant 5 s^−1^ shear rate ($\dot{\gamma })$ in comparison to samples with D water. For G8, C2.5 and A1.5 variants, viscosity decreased from 0.014 down to 0.010, from 0.496 down to 0.396 and from 0.33 down to 0.28, respectively. Viscosities of all MC hydrosols decreased during the analysis (shear rate 600–7760 s^−1^ for 2 min) while shear stress (τ) increased. The highest viscosity at minimum $\dot{\gamma }$ was measured for C2.5MC, A1.5MC, G8D and G8MC and was equal to 0.396, 0.28, 0.014 and 0.010, respectively. The differences in the rheology of hydrosols with MC and D water can be explained by the change in water structure after treatment with plasma. According to results obtained by several authors, plasma treatment causes declasterization of the water macrostructure [5,8,22]. One of the consequences is the better solubility of substances [23]. The highest shear stress at the maximum shear rate was noticed for G8MC, C2.5D, C2.5MC, A1D and A1.5MC samples. The flow behavior index (n) is an indicator of the type of fluid in which n = 1 indicates a Newtonian fluid, n < 1 a shear thinning fluid and n > 1 indicates a shear thickening or dilatant fluid. Sodium alginate and carrageenan hydrosols showed pseudoplastic behavior (n < 1), which is in agreement with Junyi et al. [24] and Król et al. [25]. The results revealed that all tested gelatin samples behave as shear-thickening fluids at high shear rates. Subhash et al. [26] made a similar observation. Additionally, it was noticed that flow behavior decreased with the increase in polymer concentration. The results showed that the consistency coefficient (k) increased with the increase of the polymers concentration, which can be due to changes in the intermolecular interactions between the polymer’s molecules [24,27]. However, the value of this parameter was lower for samples with MC water, which is due to difference in viscosity. According to Zimoch-Korzycka et al. [28], the consistency index gives an idea of the solution viscosity, and this statement is in agreement with our results. Yield stress ($\tau_{0}$) informs the strength of the structure which can be defined as a resistance of the fluid structure to deformation or breakdown [29]. If the yield stress is exceeded, the dispersion starts to flow like a low-viscosity liquid [30]. There were no significant differences between the results obtained for all gelatin samples while negative yield stress was measured for carrageenan and sodium alginate variants. The lowest yield stress was exhibited by C2.5D, C2.5MC and A1.5MC samples, which also demonstrated the highest consistency index and the lowest flow behavior index. These results can be explained by the solidification of materials caused by the high polymer concentration, which leads to inhibition of its ability to flow [30,31].

#### 2.2.2. Time-Dependent Thixotropic Behavior

Thixotropy is a time-dependent shear thinning property. This rheological characteristic gives information about hydrocolloid stability after stirring. Certain gels or fluids show a time-dependent reduction in viscosity under shear stress. In the food industry, thixotropy is used to achieve desired texture of the food by stirring or resting. The results of thixotropy areas measurement (Table 2) showed recovery of the structure of all gelatin and sodium alginate samples. However, gelatin MC variants rebuilt their structure faster than control samples, which is in contrast to sodium alginate hydrosols. The worst structure recovery was noticed for A1.25MC and G8D samples. The results revealed that the use of micro-clustered water for gelatin hydrosol preparation may better prevent runoff from the surface of the product and sedimentation of additives. The negative thixotropic area of carrageenan hydrosols proves the damage of its structure with the increase in shear rate [24]. In accordance with Benchabane and Bekkour [32], it can be also seen that the thixotropy behavior is stronger with increasing concentrations, which is due to an increase in the intermolecular interaction between molecules.

#### 2.2.3. Determination of the Viscoelastic Properties

To characterize the changes in the microstructure and stability, during storage, hydrosols were subjected to oscillating shear deformation. The results of the storage modulus (G′), loss modulus (G″) and loss tangent (δ) obtained for a frequency of 5 Hz are shown in Table 2. Storage and loss moduli were evaluated as functions of shear strain amplitude. Loss tangent is the ratio of G″/G′ which characterizes the strength of colloidal forces. When G″ > G′, then the solution indicates fluid-like behavior, while when G″ < G′, then the material shows solid- or gel-like behavior [30]. All tested hydrosols showed δ > 1, which characterizes them as liquid-like/viscous. The obtained results for the control samples are in agreement with other authors [24,30,33]. There was no significant difference between samples prepared based on micro-clustered water and distilled water. With an increase in polymer concentration, the strength of the polymer network increased as indicated in G′, G″ and viscosity. As mentioned before, the viscosity of the hydrosols increased as a result of van der Waals forces and the hydrogen bonding per unit volume [34].

### 2.3. Contact Angle Measurement

The contact angle is widely used to describe the wetting, spreading and adhesion processes between a liquid and a solid. According to Young’s Equation (1), the size of the contact angle *θ* is determined by the surface tension of the liquid (γ_LV_), the interfacial tension between a liquid and solid (γ_SL_) and the surface free energy of the solid (γ_SV_) [35]:

γ_LV_ cos *θ* = γ_SV_ − γ_SL_ (1)

**Figure 2:**
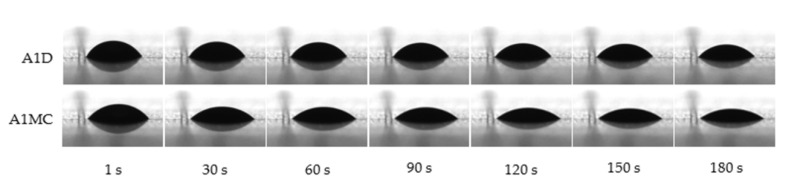
Contact angle changes during 180 s of hydrosols analysis.

In the case of complete wetting, the contact angle is close to 0°. Between 0° and 90°, the solid is wettable, and above 90° it is not wettable. Photographs of surface wetting are presented in Figure 2. The results revealed a smaller contact angle in the case of samples prepared with MC water rather than D water. After 30 s of measurement, the contact angle of A1MC and A1D was equal to 41.46° ± 0.77 and 49.893° ± 0.43, respectively (Table 3). Chen and Bonaccuro [36] found out that surface wettability does not affect the duration of inertial wetting, whereas the viscosity of the liquid does. The author suggests that the inertial wetting time increases with liquid viscosity, which is in agreement with our results. As mentioned above, the use of MC water for carrageenan and sodium alginate hydrosols’ preparation caused a significant decrease in apparent viscosity in comparison to samples with D water. According to our results, the lower the viscosity, the lower the contact angle. Moreover, according to Ismail et al. [37], also the pH of the fluid has great importance on the contact angle due to its influence on the surface electrical potential.

### 2.4. Antioxidant Activity

**Figure 3:**
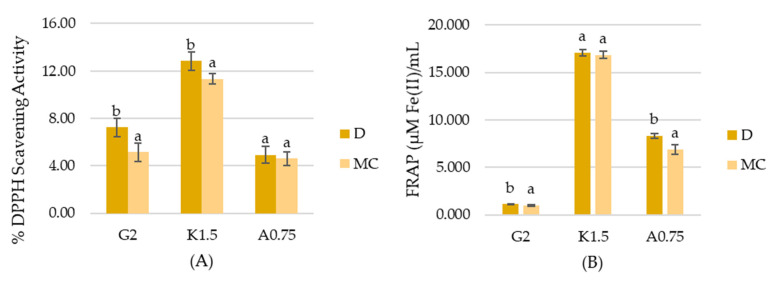
Antioxidant activity determined by (**A**) DPPH method, and (**B**) FRAP method. ^a,b^ Different letters indicate significantly different groups determined by Duncan’s test (*p* < 0.05).

The results of antioxidant activity measured with DPPH method (Figure 3A) revealed that there is a slight deterioration of the antioxidant capacity of gelatin and carrageenan hydrosols with MC water while there is no impact on the properties of sodium alginate hydrosols. K.1.5MC sample was characterized by DPPH Scavenging Activity lower than that of the control sample by only 1.5%. In the case of the gelatin sample, the antioxidant activity of G2MC was lower by 2.11%. The ferric reducing antioxidant power (FRAP) analysis (Figure 3B) provided information about the chelating ability of tested hydrosols. There was no significant difference (*p* < 0.05) between carrageenan variants. However, G2MC and A0.75MC samples showed slightly lower activity, measured by the FRAP method. The above results can be explained by the generation of strong oxidizing activity of products generated during the plasma treatment, such as oxygen, ozone (O_3_), superoxide anion (O_2_^−^), hydrogen peroxide (H_2_O_2_), hydroxyl radicals (HO) and other generating-ROS species [38,39,40]. Results obtained by Król et al. [41] showed that sodium alginate hydrosol with strong oxidizers has DPPH values equal to ~33 µM Trolox/mL, while the control sample is 74.33 µM Trolox/mL. It should be emphasized that the antioxidant activity of samples with MC is only slightly deteriorated. This may be explained by the results obtained by Sokolova et al. [42]. The authors revealed that pH has a great influence on the antioxidant capacity of polymers. The lower the pH, the higher % of inhibition of linoleic acid oxidation. Our study revealed that MC causes a decrease in the pH of hydrosols.

## 3. Materials and Methods

### 3.1. Material

Alginate FD 125 extracted from *Laminaria digitata* was purchased from Dupont GRINSTED^®^ Grinsted, Denmark. Gelatin from porcine skin (180 Bloom) was obtained from Weishardt (Graulhet, France) and ҝ-carrageenan from *Euchema cottoni* was purchased from Regis (Kraków, Poland).

### 3.2. Plasma Reatment

The treatment of water was performed using the apparatus described in [6]. The treatment time was 15 min at a pressure of 10 Pa and discharge power of 8.5 W. After 2 h devoted to relaxation, we measured an increase in pH from 6.2 to 8.1 which we assumed to be proof of treatment. The details of the mass spectrometry studies along with pH measurements of similar water samples may be found in [6].

### 3.3. Preparation of Hydrosols

Hydrosols were prepared by dissolving gelatin (G)/carrageenan (C)/sodium alginate (A) in distilled (D) or micro-clustered water (MC). Gelatin and carrageenan solutions were heated up to 60 °C and stirred at 300 rpm (IKA, RW 20 digital, Staufen m Breisgau, Germany) for 10 min. Sodium alginate was prepared at room temperature and stirred for 30 min. The composition of the prepared solution is shown in Table 4.

### 3.4. Hydrosols Characterization

#### 3.4.1. Physiochemical Properties of Hydrosols

The oxidation–reduction potential (ORP), pH and electrical conductivity (EC) of hydrosols prepared on the basis of D and MC water were measured using a pH/mV/ISE Meter (Seven Multi™ model S40, Mettler Toledo, Warsaw, Poland) equipped with a pH electrode (Inlab Routine Pro, Mettler Toledo), ORP electrode (Inlab Redox Pro, Mettler Toledo) and conductivity electrode (InlabLab 731, Mettler Toledo), respectively.

#### 3.4.2. Rheological Measurements

##### Flow Property

The flow properties were tested using HAAKE RheoStress 6000 rheometer (Thermo Scientific, Karlsruhe, Germany) at 25 °C for sodium alginate, 35 °C for gelatin and 45 °C for carrageenan. A cone sensor (C60/1° Ti L, Thermo Scientific, Karlsruhe, Germany) and measuring plate (TMP60 Steel 18/8, Thermo Scientific, Karlsruhe, Germany) in CS mode were used. The shear stress (τ) and viscosity (η) measurements were obtained at a constant shear rate of 5 s^−1^ within 2 min and under a controlled shear rate of 600–7760 s^−1^ within 2 min. The Herschel–Bulkley model (Equation (2)) and Ostwald de Waele (Equation (3)) model were used, as follows:

(2) $$\tau =K{\dot{\gamma }}^{n},$$

(3) $$\tau =\tau_{0}+K{\dot{\gamma }}^{n}$$

where τ is the shear stress (Pa), $\dot{\gamma }$ is the shear rate ($s^{-1}$), K is the consistency index (Pa$\cdot s^{n}$), *n* is the flow behavior index (dimensionless), and $\tau_{0}$ is the yield stress (Pa).

##### Time-Dependent Thixotropic Behavior

Ramped-up time- and ramped-down time-flow curves were used to determine the time-dependent shear thinning property. The hysteresis loop was obtained by increasing the shear rate from 0 s^−1^ to 100 s^−1^ for 2 min, followed by 1 min at maximum shear rate (100 s^−1^) and decreasing in 2 min shear rate from 100 s^−1^ to 0 s^−1^. The thixotropic area is the hysteresis area between the increasing and decreasing shear rate curves and was calculated using RheoWin Data Manager software version 4.00, Karlsruhe, Germany.

##### Determination of the Viscoelastic Properties

The viscoelastic properties were tested using an oscillatory shear test. A strain sweep of 0.001–10 Pa at a frequency of 1 Hz was used to determine the stress range for linear viscoelasticity. Frequency sweep measurements were evaluated over a range of oscillatory frequencies 0.01–10 Hz at a constant strain oscillation amplitude (0.01 Pa). The storage modulus (G′), loss modulus (G″), and loss tangent (δ) were recorded as functions of variable frequency.

#### 3.4.3. Contact Angle Measurements

The contact angle measurements were performed with a goniometer DSA 25E (Kruss, DSA 100 Hamburg, Germany). The hydrosol droplet was gently placed on the surface of quartz plate by a glass syringe with a needle of diameter 0.5 mm. The liquids were dispensed in a volume of 2.0 µL with a flow rate of 2.67 µL/s. The process was recorded with a high-speed camera (FASTCAM SA-1, Photron, Inc., Tokyo, Japan) with recording rates of 10,000–53,000 per second. One-percent sodium alginate hydrosols were used for the experiment.

#### 3.4.4. Antioxidant Activity

##### Free Radical Scavenging Activity (DPPH)

Free radical scavenging activity of the G2D/G2MC, C1.5D/C1.5MC and SA0.75D/SA0.75MC was determined by the method of Chen et al. [43]. One milliliter of hydrosol was incubated with 0.1 mM DPPH (2.2-diphenyl-1-picrylhydrazyl)-methanol solution for 30 min. The reduction in the DPPH free radicals was measured by reading the absorbance at 517 nm. The percentage of the radical scavenging activity (RSA) was calculated using the following equation based on Olugbami et al. (2015) [44].

DPPH Scavenged (%) = (A_control_ − A_sample_)/A_control_ × 100% (4)

where A_control_ and A_sample_ are the absorbance values (at 517 nm) for the control and sample, respectively.

##### Ferric Reducing Ion Antioxidant Power (FRAP)

The method described by Benzie and Strain [45] was used to determine the antioxidant power of hydrosols. Three mL of FRAP reagent was mixed with 100 uL of hydrosol. The absorbance was read at 593 nm. Standard calibration solutions were prepared with ferrous sulfate (0–1 mM).

### 3.5. Statistical Analysis

The statistical analysis was performed with multivariate analysis of variance (ANOVA) using Statistica 10 (StatSoft, Cracow, Poland). The differences between the mean values were identified by the Duncan Test with a confidence level at *p* < 0.05. All of the experiments were performed in triplicate.

## Figures and Tables

**Table 1:** Flow properties of hydrosols.

Variant	Ostwald De Waele Model		Herschel–Bulkley Model	Shear Stress τ_5_fl (Pa)	Shear Stress τ_7760_ (Pa)	Apparent Viscosity η_5_ (Pa∙s)	Apparent Viscosity η_7760_ (Pa∙s)
	Consistency Index *k* (Pa∙s)	Flow Behavior Index *n* (-)	Yield Stress τ_0_ (Pa)				
G2D	0.13 ^a^ ± 0.08	1.33 ^c^ ± 0.07	0.05 ^a^ ± 0.03	0.04 ^a,b^ ± 0.02	19.05 ^a^ ± 5.65	0.008 ^a,b^ ± 0.00	0.002 ^a^ ± 0.00
G4D	0.88 ^a,b^ ± 0.01	1.16 ^b^ ± 0.01	0.05 ^a^ ± 0.00	0.04 ^a,b^ ± 0.02	28.10 ^b^ ± 1.57	0.009 ^a,b^ ± 0.00	0.004 ^b^ ± 0.00
G8D	7.45 ^c^ ± 1.00	1.03 ^a^ ± 0.01	0.03 ^a^ ± 0.00	0.07 ^b^ ± 0.00	78.44 ^c^ ± 3.85	0.014 ^b^ ± 0.00	0.010 ^c^ ± 0.00
G2MC	0.15 ^a^ ± 0.01	1.31 ^c^ ± 0.00	0.04 ^a^ ± 0.00	0.01 ^a^ ± 0.00	18.76 ^a^ ± 1.30	0.002 ^a^ ± 0.00	0.002 ^a^ ± 0.00
G4MC	1.93 ^b^ ± 0.31	1.08 ^a^ ± 0.01	0.04 ^a^ ± 0.00	0.04 ^a,b^ ± 0.01	31.61 ^b^ ± 1.94	0.009 ^a,b^ ± 0.00	0.004 ^b^ ± 0.00
G8MC	6.12 ^c^ ± 1.29	1.06 ^a^ ± 0.02	0.04 ^a^ ± 0.01	0.05 ^b^ ± 0.00	87.31 ^d^ ± 0.23	0.010 ^b^ ± 0.00	0.011 ^c^ ± 0.00
C1.5D	0.82 ^a,b^ ± 0.09	0.53 ^b^ ± 0.03	−2.25 ^a^ ± 0.49	0.43 ^a^ ± 0.03	83.58 ^a^ ± 16.06	0.085 ^b^ ± 0.01	0.009 ^a^ ± 0.00
C2.0D	2.16 ^b^ ± 0.01	0.48 ^b^ ± 0.01	−6.05 ^a^ ± 0.07	1.28 ^d^ ± 0.03	162.69 ^e^ ± 0.46	0.257 ^d^ ± 0.01	0.015 ^c^ ± 0.00
C2.5D	4.60 ^c^ ± 0.12	0.39 ^a^ ± 0.01	−33.83 ^b^ ± 7.92	2.48 ^e^ ± 0.67	116.20 ^c^ ± 2.94	0.496 ^f^ ± 0.13	0.021 ^e^ ± 0.00
C1.5MC	0.73 ^a^ ± 0.03	0.53 ^b^ ± 0.02	−1.99 ^a^ ± 0.29	0.41 ^b^ ± 0.04	80.08 ^b^ ± 22.16	0.081 ^a^ ± 0.01	0.012 ^b^ ± 0.00
C2.0MC	1.74 ^a,b^ ± 0.15	0.50 ^b^ ±0.00	−4.83 ^a^ ± 0.44	1.02 ^c^ ± 0.07	146.57 ^d^ ± 7.19	0.204 ^c^ ± 0.01	0.016 ^c^ ± 0.00
C2.5MC	4.64 ^c^ ± 1.08	0.41 ^a^ ± 0.02	−28.95 ^b^ ± 16.32	1.98 ^f^ ± 0.22	120.62 ^c^ ± 6.25	0.396 ^e^ ± 0.04	0.019 ^d^ ± 0.00
A0.75D	0.93 ^a^ ± 0.06	0.54 ^a^ ± 0.00	−2.49 ^a^ ± 0.17	0.44 ^b^ ± 0.01	120.90 ^b^ ± 7.56	0.09 ^b^ ± 0.00	0.016 ^a^ ± 0.00
A1D	2.15 ^b^ ± 0.17	0.49 ^b^ ± 0.00	−6.52 ^a^ ± 0.59	0.94 ^d^ ± 0.00	173.25 ^c^ ± 0.00	0.19 ^d^ ± 0.00	0.021 ^b^ ± 0.00
A1.5D	3.80 ^c^ ± 0.04	0.45 ^c^ ± 0.00	−14.36 ^a^ ± 0.42	1.63 ^f^ ± 0.00	181.13 ^c^ ± 40.09	0.33 ^f^ ± 0.03	0.023 ^b^ ± 0.01
A0.75MC	0.86 ^a^ ± 0.02	0.55 ^a^ ± 0.00	−2.33 ^a^ ± 0.07	0.41 ^a^ ± 0.01	113.24 ^a,b^ ± 4.17	0.08 ^a^ ± 0.01	0.009 ^a^ ± 0.00
A1MC	3.90 ^c^ ± 0.04	0.38 ^d^ ± 0.00	−39.63 ^a,b^ ± 1.03	0.77 ^c^ ± 0.00	70.02 ^a^ ± 1.59	0.15 ^c^ ± 0.00	0.015 ^a^ ± 0.00
A1.5MC	5.27 ^d^ ± 0.25	0.39 ^d^ ± 0.03	−63.61 ^b^ ± 44.83	1.38 ^e^ ± 0.02	117.95 ^b^ ± 27.71	0.28 ^e^ ± 0.04	0.014 ^a^ ± 0.00

^a–f^ Values with different letters within the same column differ significantly (*p* < 0.05).

**Table 2:** Frequency sweep values and thixotropy area of hydrosols.

Variant	Storage Modulus G′_5Hz_ (mPa)	Loss Modulus G″_5 Hz_ (mPa)	Loss Tangent Tanδ_5Hz_ (Pa)	Thixotropy Area (Pa/s)
G2D	3.69 ^a^ ± 2.79	13.50 ^a^ ± 9.20	6.45 ^a^ ± 7.38	0.38 ^a^ ± 0.01
G4D	3.98 ^a^ ± 0.35	14.95 ^a^ ± 0.88	3.78 ^a^ ± 0.55	4.15 ^d^ ± 0.72
G8D	1.43 ^a^ ± 0.03	55.86 ^b^ ± 0.68	39.00 ^b^ ± 0.43	6.17 ^e^ ± 2.89
G2MC	6.47 ^a^ ± 0.07	8.04 ^a^ ± 0.01	1.24 ^a^ ± 0.01	0.70 ^a^ ± 2.59
G4MC	6.16 ^a^ ± 2.17	16.63 ^a^ ± 1.10	2.90 ^a^ ± 1.21	1.49 ^b^ ± 0.24
G8MC	3.65 ^a^ ± 3.56	119.81 ^b^ ± 32.67	16.53 ^b^ ± 1.18	2.76 ^c^ ± 0.02
C1.5D	9.85 ^a^ ± 1.97	398.73 ^a^ ± 5.99	41.35 ^b^ ± 8.86	−1.22 ^a^ ± 0.07
C2.0D	58.18 ^b^ ± 16.26	1071.74 ^b,c^ ± 148.97	18.80 ^a^ ± 2.69	−6.31 ^c^ ± 0.13
C2.5D	195.80 ^c^ ± 17.21	2760.00 ^d^ ± 213.39	14.10 ^a^ ± 0.15	−20.25 ^d^ ± 0.74
C1.5MC	10.49 ^a^ ± 2.18	476.18 ^a,b^ ± 86.67	45.50 ^b^ ± 1.18	−5.62 ^b^ ± 3.41
C2.0MC	63.91 ^b^ ± 3.91	1205.89 ^c^ ± 31.74	18.92 ^a^ ± 1.65	−6.41 ^c^ ± 6.65
C2.5MC	149.58 ^c^ ± 36.17	2257.76 ^d^ ± 559.69	15.08 ^a^ ± 0.09	−30.20 ^e^ ± 40.00
A0.75D	9.88 ^a^ ± 7.87	560.10 ^a^ ± 47.32	34.68 ^b^ ± 0.79	0.76 ^b^ ± 0.05
A1D	29.37 ^b^ ± 1.01	1144.00 ^c^ ± 4.45	38.97 ^b^ ± 1.19	1.71b ^c^ ± 0.00
A1.25D	86.27 ^d^ ± 4.44	2083.61 ^e^ ± 168.92	24.14 ^a^ ± 0.72	1.91 ^c^ ± 0.01
A0.75MC	2.80 ^a^ ± 0.16	457.13 ^a^ ± 3.86	63.75 ^c^ ± 7.82	0.64 ^a^ ± 0.00
A1MC	22.32 ^b^ ± 0.87	904.22 ^b^ ± 30.06	40.52 ^b^ ± 0.23	3.26 ^d^ ± 1.29
A1.25MC	53.08 ^c^ ± 2.68	1394.64 ^d^ ± 51.09	26.28 ^a^ ± 0.36	10.72 ^e^ ± 1.63

^a–e^ Values with different letters within the same column differ significantly (*p* < 0.05).

**Table 3:** Contact angle data of hydrosols.

Variant	Time of Measurement (s)	Water Contact Angle (°)
A1D	**1**	49.89 ^b^ ± 0.73
A1MC		44.29 ^a^ ± 0.80
A1D	**30**	49.89 ^b^ ± 0.43
A1MC		41.46 ^a^ ± 0.77
A1D	**60**	48.36 ^b^ ± 0.55
A1MC		38.28 ^a^ ± 0.41
A1D	**90**	46.8 2 ^b^ ± 0.55
A1MC		37.43 ^a^ ± 0.46
A1D	**120**	45.07 ^b^ ± 0.26
A1MC		36.43 ^a^ ± 0.56
A1D	**150**	44.51 ^b^ ± 0.36
A1MC		36.01 ^a^ ± 0.74
A1D	**180**	41.07 ^b^ ± 0.11
A1MC		30.41 ^a^ ± 0.55

^a,b^ Values with different letters within the same column differ significantly (*p* < 0.05).

**Table 4:** The composition of hydrosols.

Run Code Letters			Gelatin (G) [%]	Carrageenan (C) [%]	Sodium Alginate (A) [%]	Water (D or MC)
G2D	C1.5D	A0.75D	2.0	1.5	0.75	D
G2MC	C1.5MC	A0.75MC				MC
G4D	C2D	A1D	4.0	2.0	1.0	D
G4MC	C2MC	A1MC				MC
G8D	C2.5D	A1.25D	8.0	2.0	1.25	D
G8MC	C2.5MC	A1.25MC				MC

## Data Availability

The data presented in this study are available on request from the corresponding author.
